# The application of traditional machine learning and deep learning with EEG for mild cognitive impairment: a bibliometric analysis

**DOI:** 10.3389/fnagi.2026.1880346

**Published:** 2026-08-03

**Authors:** Zaolv Wan, Zhangxin Lin, Yina Liu, Ruyi Wang, Ruiting Yang, Yating Ai

**Affiliations:** 1 School of Nursing, Hubei University of Chinese Medicine, Wuhan, China; 2 Hubei Shizhen Laboratory, Wuhan, China

**Keywords:** Alzheimer’s disease, bibliometric analysis, deep learning, electroencephalography, machine learning, mild cognitive impairment

## Abstract

**Objective:**

This study uses bibliometric methods to analyze the status of electroencephalography (EEG) and machine learning applications in mild cognitive impairment (MCI) research.

**Methods:**

Relevant literature was searched in the four major English databases (Web of Science, PubMed, IEEE Xplore, and Scopus) and the three major Chinese databases (CNKI, Wanfang, and VIP) from their inception to June 2026. After removing duplicates using NoteExpress and screening the literature according to the inclusion and exclusion criteria, the final data were imported into CiteSpace 6.4. R2 and VOSviewer 1.6.20 for analysis of publication trends, geographic distribution, highly co-cited references, keyword clustering, and burst detection.

**Results:**

A total of 547 valid studies were included in the analysis, comprising 523 English-language studies and 24 Chinese core journal articles. The number of publications in this field has shown phased growth. It entered a period of rapid development after 2018. China ranks first globally in terms of English-language publications; however, there is a significant gap between the number of core publications in Chinese and English. *Clinical Neurophysiology* is the journal with the highest total local citation score (TLS). Four of the top 10 highly co-cited journals are published in the United States. Highly cited literature has jointly contributed to establishing a core body of knowledge in this field. This knowledge encompasses clinical diagnostic guidelines, electrophysiological mechanisms, signal processing methods, and intelligent algorithm models. Traditional machine learning keywords focus on methodological exploration, including feature extraction and support vector machine classification. Deep learning keywords focus on model applications, including convolutional and artificial neural networks. Keyword burst analysis identifies feature selection, decision trees, and neuropsychological assessment as the current frontier topics in this field.

**Conclusion:**

Electroencephalography combined with machine learning has become an important research direction for the early identification and assessment of MCI. The research focus has shifted from traditional EEG signal analysis and manual feature engineering to optimization of deep learning models and exploration of multimodal data fusion. Improved algorithm interpretability, refined EEG rhythm and brain region localization, and the combination of neuropsychological assessment and EEG indicators have become a new research trend.

## 1 Introduction

Alzheimer’s disease (AD) is one of the leading causes of disability and death in the elderly population worldwide. Currently, there is no cure, and early intervention is the only effective strategy to delay disease progression (Scheltens et al., 2021). Mild cognitive impairment (MCI) is the only modifiable key transitional stage between normal aging and AD. Its core clinical manifestations are cognitive decline and memory loss. Activities of daily living are not significantly affected. The annual conversion rate to dementia among MCI patients is as high as 10–15%, which is much higher than that of the general elderly population (Bradfield, 2023; Vega and Newhouse, 2014). Epidemiological data show that the prevalence of MCI among adults aged 60 years and older worldwide is 6.7–25.2% (Jongsiriyanyong and Limpawattana, 2018). The overall prevalence among individuals aged 55 years and older in China is reported to be 15.4% (Deng et al., 2021). With the intensification of global population aging, MCI has become a major public health issue. Accurately identifying MCI early and predicting its progression represents the most cost-effective critical window for intervention to block AD onset and reduce dementia burden.

Electroencephalography (EEG), a non-invasive, low-cost neurophysiological detection technique with high temporal resolution, can capture characteristic abnormalities in brain network oscillations in patients with MCI (Wijaya et al., 2023). EEG is regarded as a potential objective electrophysiological biomarker for the early identification of the disease (Jiang et al., 2025). However, traditional EEG analysis relies heavily on manual interpretation by experts, which can lead to several limitations. These include strong subjectivity, difficulty in capturing weak non-linear abnormalities, and limited diagnostic accuracy (Aanestad et al., 2024). Traditional machine learning methods, such as support vector machine (SVM) and random forest, were first applied to address these challenges. These methods construct classification models by manually extracting EEG spectrum and non-linear dynamic features, and related studies have initially explored the automatic identification of cognitive impairment (Chriskos et al., 2025). However, the performance of traditional machine learning methods is heavily dependent on the expertise in feature engineering. These methods have difficulty in mining high-dimensional hidden information. The development of deep learning technology provides a potential approach to address this limitation. Related studies aim to automatically extract high-dimensional hidden features from EEG signals in an end-to-end manner and explore the accurate classification of MCI and predict the risk of conversion (Craik et al., 2019; Franzmeier et al., 2017). In recent years, traditional machine learning and deep learning have developed in parallel, jointly driving the rapid growth of EEG-based MCI research. This field has become a hotspot intersecting neuroscience, medical artificial intelligence, and geriatrics.

Bibliometrics is a mature interdisciplinary field that quantitatively analyzes the development characteristics, hotspots, and trends within various disciplines using mathematical and statistical methods. When combined with information visualization technology, it can intuitively and comprehensively present the knowledge structure and evolutionary patterns of a field (Chen et al., 2021). Currently, CiteSpace and VOSviewer are the most widely used analytical tools for visualizing bibliometric information. These tools can efficiently realize core analyses such as cooperation networks, co-citation analysis, topic clustering, and frontier bursts. They have been extensively validated and applied across many interdisciplinary medical fields.

To date, a small number of studies have conducted bibliometric analyses of the application of EEG and artificial intelligence in cognitive impairment. However, the majority of existing studies generally cover the application of various artificial intelligence technologies across the full course of AD. They have not focused on the precise interdisciplinary field of combining machine learning with EEG for the diagnosis of MCI. Moreover, the vast majority of studies only include English databases such as Web of Science or PubMed, thus ignoring research findings from Chinese databases and failing to fully reflect the research contributions and characteristics of developing countries, including China.

Accordingly, this study included relevant literature from three major Chinese databases. These are China National Knowledge Infrastructure (CNKI), Wanfang, and VIP. It also included four major English databases. These are Web of Science Core Collection, PubMed, IEEE Xplore, and Scopus. The search period covered from the establishment of these databases to June 2026. Using bibliometric methods, this study conducted a systematic visual analysis using CiteSpace and VOSviewer software to provide references for subsequent research in this field.

## 2 Materials and methods

### 2.1 Data sources

This study searched four English databases (Web of Science Core Collection, PubMed, IEEE Xplore, Scopus) and three Chinese databases (CNKI, Wanfang Data, VIP Database). Covering multiple disciplines, including medicine, computer science, and neuroscience, these databases ensure the comprehensiveness and representativeness of the literature search. The search period was set from the inception of each database to 5 June 2026.

### 2.2 Search strategy

Boolean logical operators (AND/OR) were uniformly applied to construct relationships between and within conceptual groups. The right truncation symbol (*) was used to cover plural forms, word variants, and common abbreviations of core search terms, thereby minimizing literature omission to the maximum extent. For Chinese databases, searches were conducted using the subject field (SU).

English search strategy: TS = (“Mild Cognitive Impairment” OR MCI OR “Cognitive Dysfunction” OR “pre-dementia” OR “early Alzheimer’s”) AND TS = (“Electroencephalography” OR EEG OR “Electroencephalogram” OR “brain wave*” OR “EEG signal*”) AND TS = (“Deep Learning” OR “Machine Learning” OR “Artificial Intelligence” OR “Neural Network*” OR “Support Vector Machine*” OR SVM OR “Convolutional Neural Network*” OR CNN OR “Recurrent Neural Network*” OR RNN OR LSTM OR “Deep Neural Network*” OR DNN).

Chinese search strategy: (SU = ‘轻度认知障碍’ OR SU = ‘认知功能障碍’ OR SU = ‘MCI’ OR SU = ‘遗忘型轻度认知障碍’ OR SU = ‘aMCI’) AND (SU = ‘脑电图’ OR SU = ‘脑电’ OR SU = ‘脑电信号’ OR SU = ‘EEG’) AND (SU = ‘深度学习’ OR SU = ‘深度神经网络’ OR SU = ‘机器学习’ OR SU = ‘卷积神经网络’ OR SU = ‘CNN’ OR SU = ‘循环神经网络’ OR SU = ‘RNN’ OR SU = ‘LSTM’).

### 2.3 Inclusion and exclusion criteria

Inclusion criteria included: (1) study subjects included individuals with MCI, and all included studies adopted a control design, covering two-group comparisons of MCI and healthy controls, as well as multi-group comparisons of MCI, AD/other cognitive diseases, and healthy controls and (2) electroencephalogram/electroencephalographic signals served as core data, with deep learning or machine learning algorithms applied for analysis.

Exclusion criteria included: reviews, duplicate publications, and literature failing to meet the above inclusion criteria.

### 2.4 Literature screening and data processing

**Figure 1:**
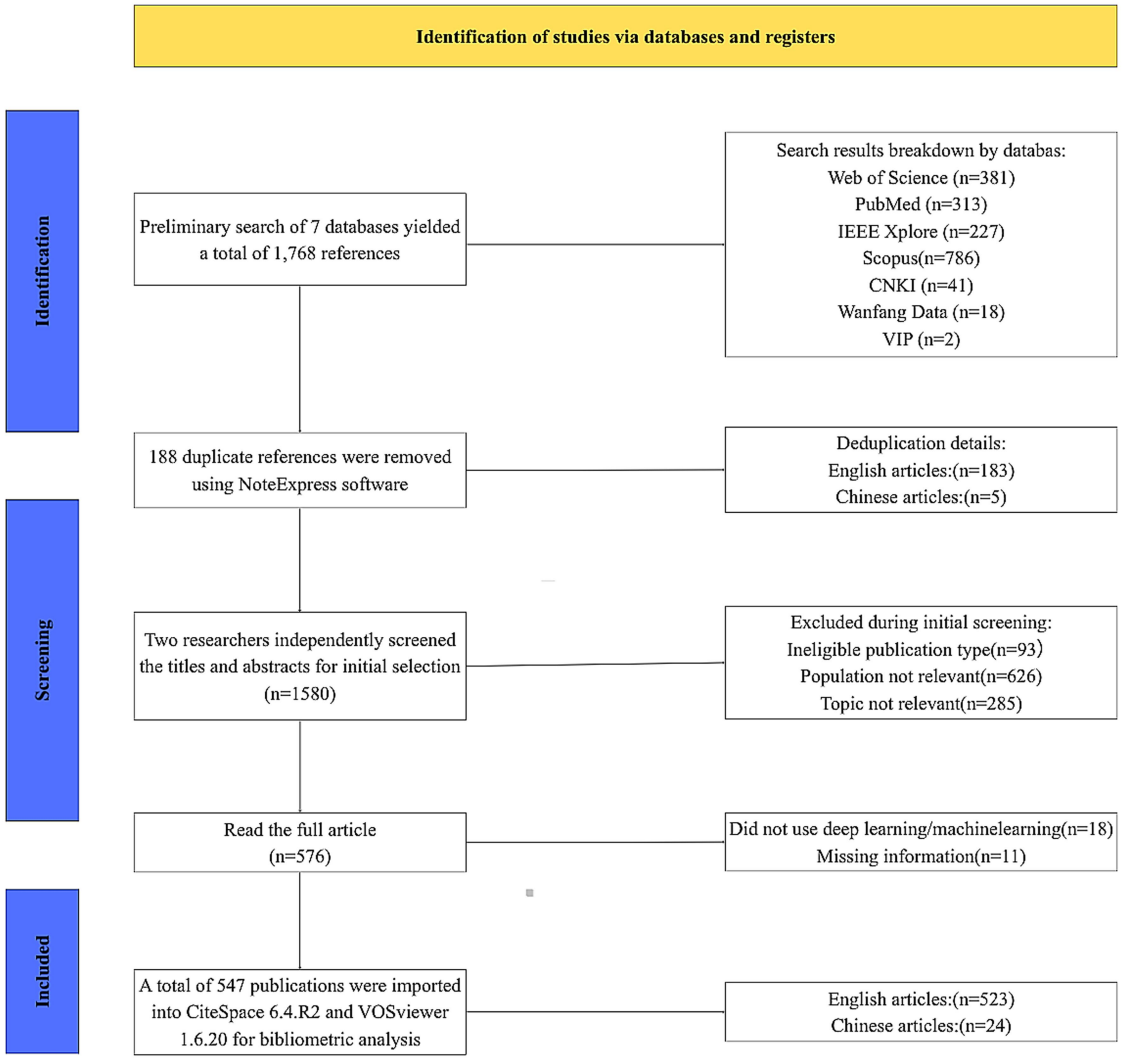
Flowchart of literature screening.

Two researchers independently screened the literature. First, search results from all databases were collectively imported into NoteExpress 3.6 software. After removing duplicate records, they initially excluded literature that clearly did not meet the inclusion criteria by reviewing titles and abstracts. Then, the remaining literature was manually checked via full-text reading to complete re-screening. Any screening discrepancies were resolved through mutual discussion to reach consensus. Unresolved disagreements were adjudicated by a third researcher. The screened Chinese literature was exported in RefWorks format, while English literature was exported in plain text format. We then performed unified data standardization, extracting fields such as title, author, affiliation, year, keywords, and references and standardizing terminology and institution names accordingly. Finally, a total of 547 valid documents were included, and bibliometric and visual analyses were carried out using CiteSpace 6.4.R2 and VOSviewer 1.6.20 (Figure 1).

## 3 Results

### 3.1 Status of literature publications

**Figure 2:**
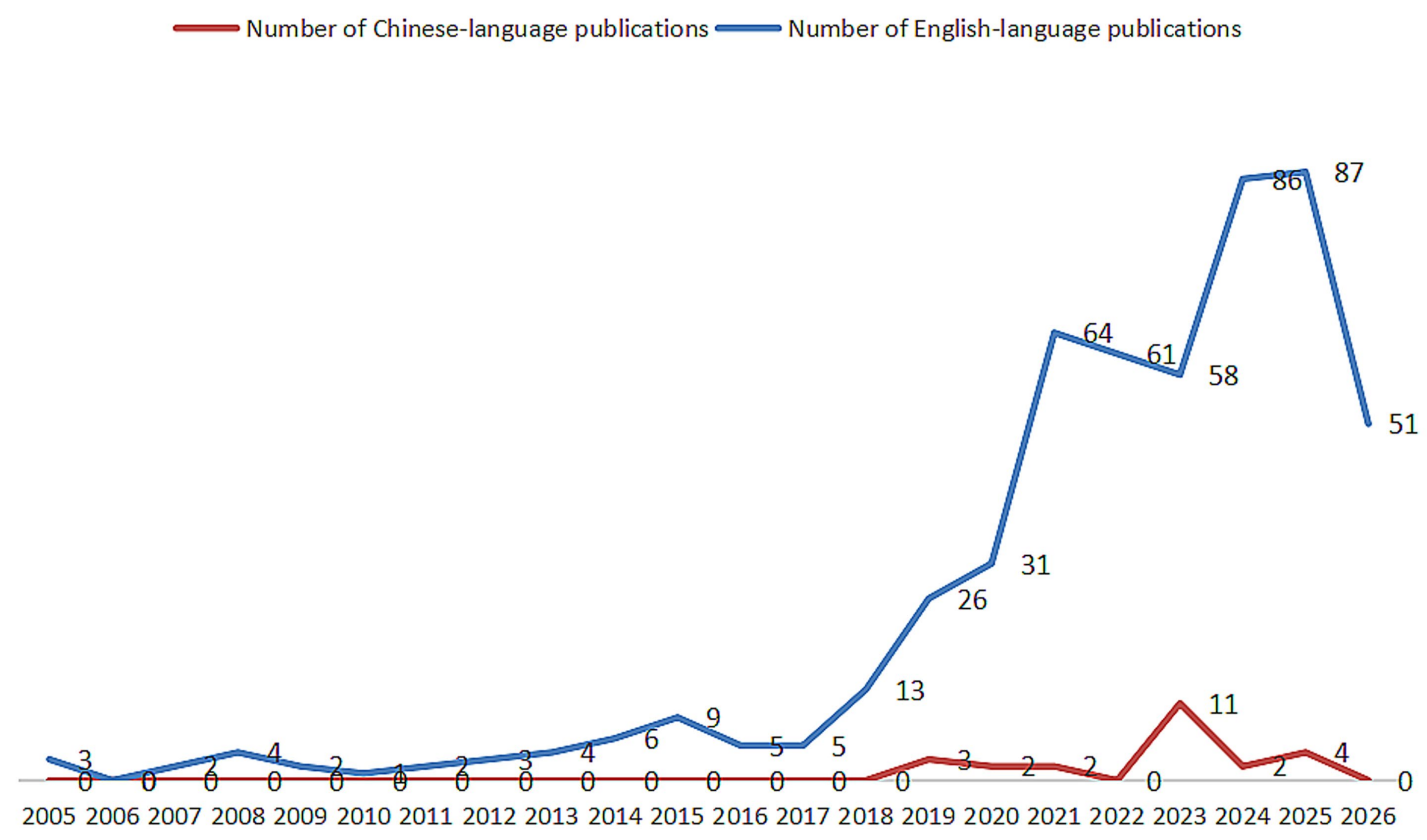
Annual publication trend of machine learning in EEG for MCI.

Figure 2 shows the publication output on machine learning using electroencephalography in the field of MCI. The year is the abscissa. The annual number of publications is the ordinate. It can be seen that the number of publications remained low from 2005 to 2017. After 2018, the field entered a stage of steady growth, with annual output gradually increasing. In 2021, it entered a phase of high-level fluctuation. It reached peaks of 86 articles in 2024 and 87 articles in 2025, suggesting that this interdisciplinary research field has attracted widespread attention from the academic community.

It is worth noting that only 24 relevant Chinese papers were retrieved from China’s three major core databases. Research in this field started relatively late in China. The number of publications peaked at 11 in 2023. This stands in sharp contrast to the high output of 137 articles published by Chinese scholars in international English journals.

### 3.2 Geographical distribution of publications

**Figure 3:**
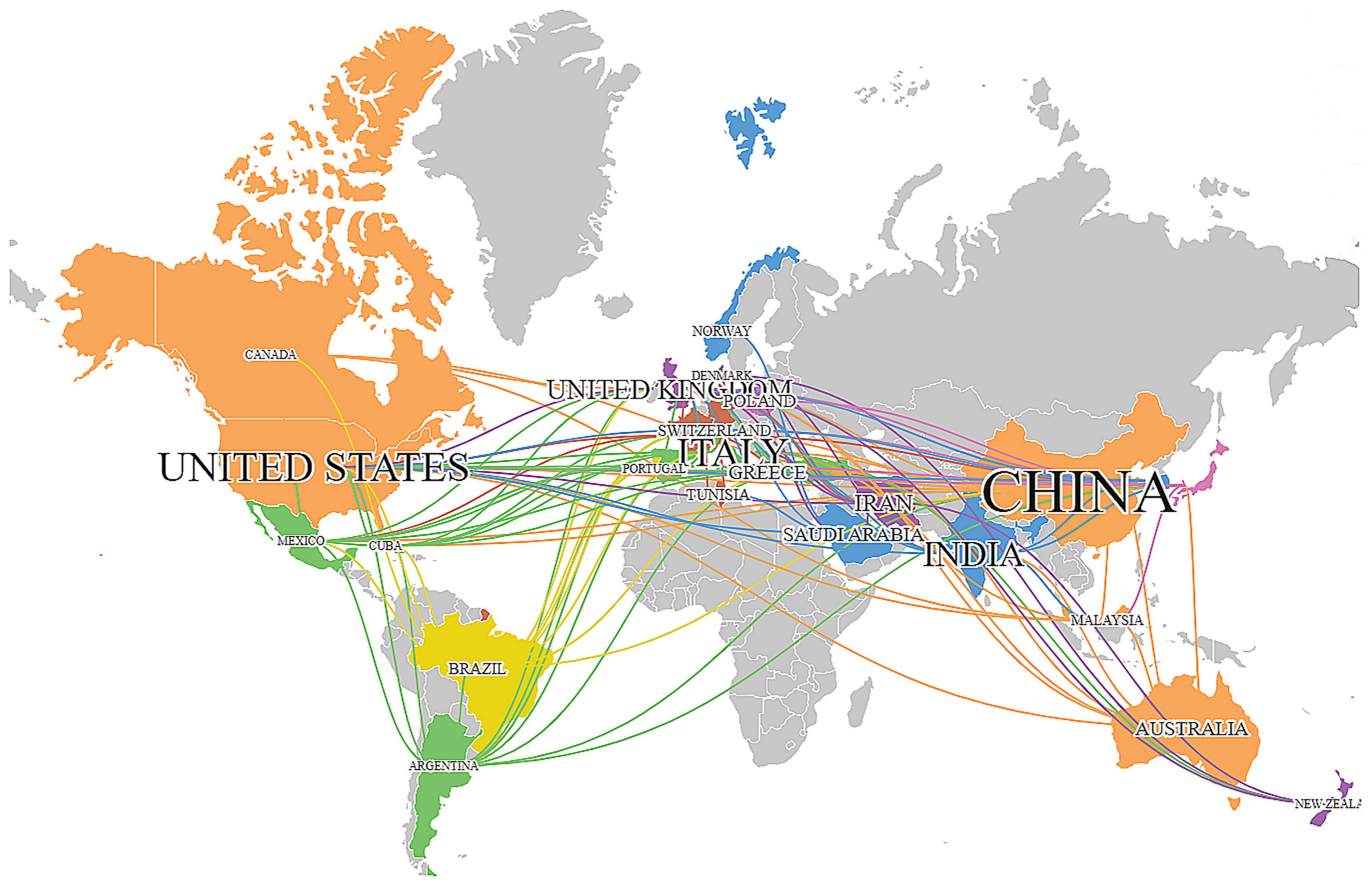
Geographical map of national research collaboration network.

Figure 3 is a geographic visualization map of global research cooperation. It was generated using Scimago Graphica software. Node size represents the number of publications by country. Line thickness indicates the strength of cooperation between countries. Different colors represent distinct research cooperation clusters. The results show that research outputs are mainly concentrated in East Asia, North America, and Europe. Table 1 lists the top 10 countries by publication volume. China ranks first with 137 papers (26.2%), followed by the United States (65 papers, 12.4%) and Italy (55 papers, 10.5%), indicating that China holds an important position in global research output.

**Table 1:** Top 10 countries by publication volume.

Country	Count	Percentage
China	137	26.198
USA	65	12.428
Italy	55	10.516
India	47	8.987
South Korea	37	7.075
Japan	37	7.075
United Kingdom	28	5.353
Turkey	22	4.207
Iran	21	4.015
Spain	20	3.824

### 3.3 Analysis of highly co-cited journals

**Figure 4:**
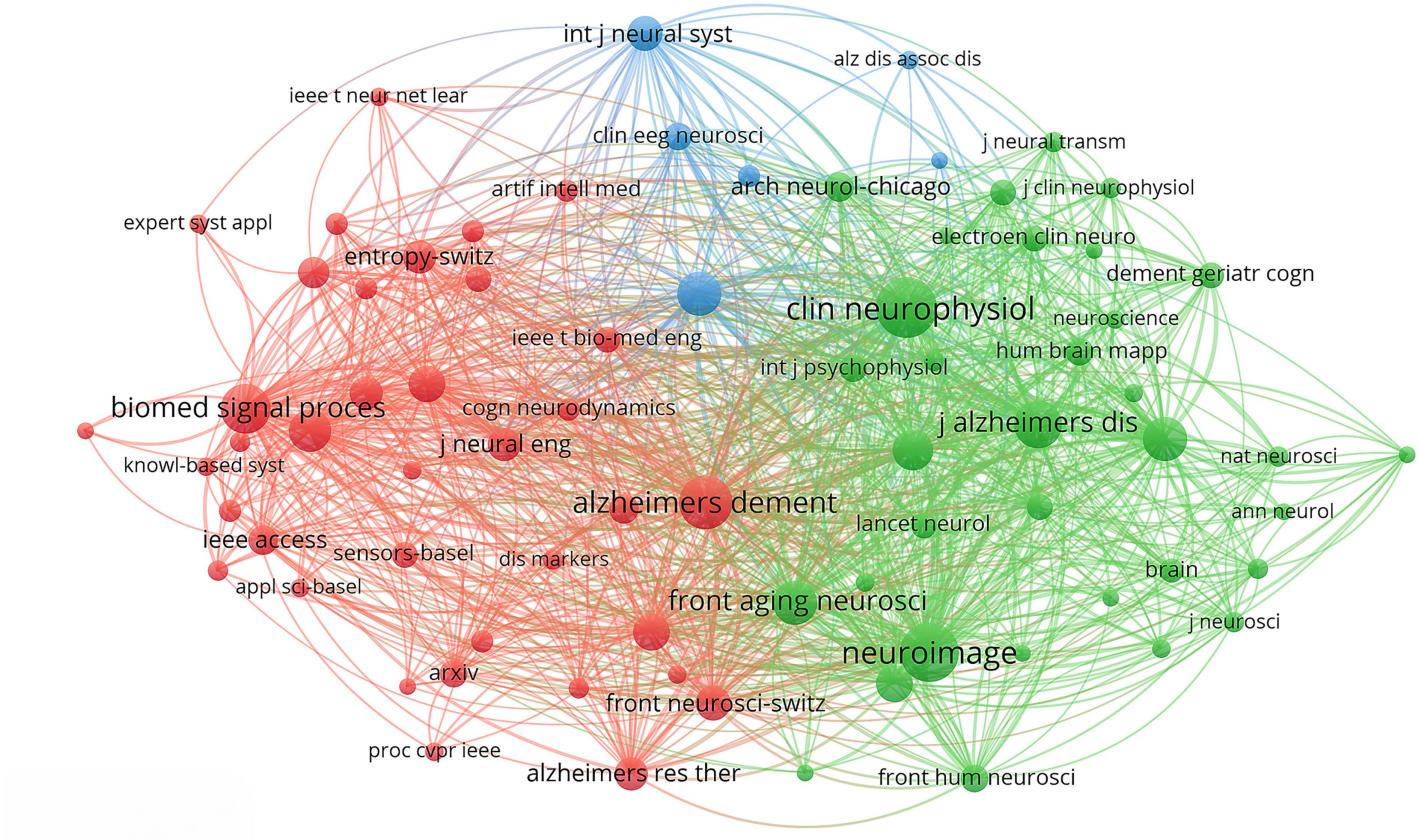
Visualization map of highly co-cited journals based on VOSviewer.

Figure 4 is a visualization map of journal co-citation. It was generated using VOSviewer software. The map shows three core research clusters: the red cluster centers on biomedical signal processing and intelligent algorithms, the green cluster focuses on clinical neuroscience and cognitive impairment research, and the blue cluster emphasizes methodological support for neuroscience. Among them, *Alzheimer’s & Dementia*, *Clinical Neurophysiology*, and *Journal of Neuroscience Methods* are the core nodes of each cluster, respectively.

Table 2 lists the top 10 highly co-cited journals. They are ranked by Total Link Strength (TLS). TLS is a key indicator for quantifying the core position of nodes in academic networks. It represents the weighted sum of the connection strengths from a node to all other nodes. A higher value indicates a wider radiation range and greater academic influence (Donthu et al., 2021). The results show that *Clinical Neurophysiology* ranks first with a TLS value of 9,007. It is the most central academic publication in this field. Although *Alzheimer’s & Dementia* ranks only fourth in publication volume, its impact factor of 11.1 is significantly higher than that of other journals. This observation reflects its prominent academic influence within this research domain. Four of the top 10 highly co-cited journals are published in the United States, and most of the rest are from European countries, reflecting a high degree of publication concentration in Europe and the United States and their academic dominance in this field.

**Table 2:** Top 10 highly co-cited journals ranked by TLS.

Journal	Impact factor	Host country	TLS
Clinical Neurophysiology	3.6	Ireland	9,007
NeuroImage	4.5	United States	8,456
Journal of Alzheimer’s Disease	3.1	Netherlands	6,307
Alzheimer’s & Dementia	11.1	United States	4,585
Neurobiology of Aging	3.5	United Kingdom	6,500
Frontiers in Aging Neuroscience	4.5	Switzerland	5,750
Biomedical Signal Processing and Control	4.9	England	4,896
Journal of Neuroscience Methods	2.3	Netherlands	4,226
Neurology	9.0	United States	3,409
IEEE Transactions on Neural Systems and Rehabilitation Engineering	5.2	United States	4,013

### 3.4 Analysis of highly co-cited references

**Figure 5:**
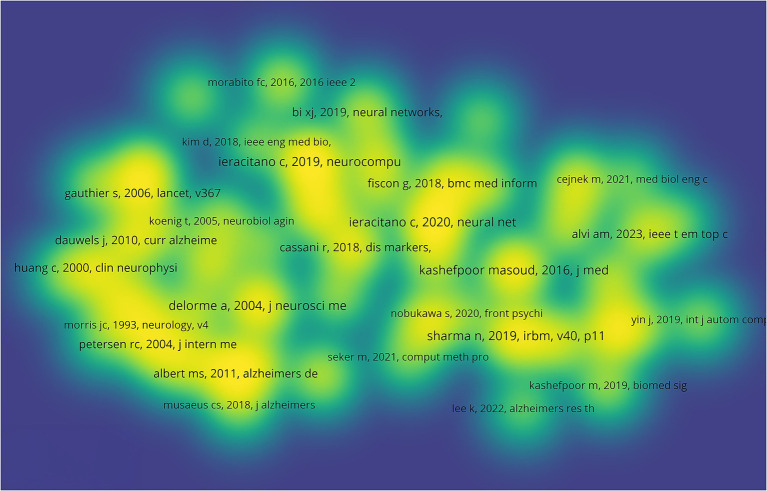
Density view of the co-cited reference network: Yellow areas represent the hotspots with the densest literature distribution.

Highly co-cited references constitute the core knowledge foundation of the field. Table 3 lists the top 20 highly co-cited documents. These have the highest TLS in the dataset of this study. Combined with the network density view in Figure 5 (yellow areas represent core knowledge clusters), it can be seen that studies on EEG signal processing methods (Delorme and Makeig, 2004), multimodal machine learning for EEG classification (Ieracitano et al., 2020) and the application of deep learning models in EEG-based cognitive impairment recognition (Ieracitano et al., 2019) have emerged as core, highly cited works in recent years.

**Table 3:** Top 20 highly co-cited references by TLS.

No.	Reference title	Type	Year	TLS
1	A novel multi-modal machine learning-based approach for automatic classification of EEG recordings in dementia	Review	2020	297
2	Automatic diagnosis of mild cognitive impairment using electroencephalogram spectral features	Article	2016	282
3	EEG and cognitive biomarkers based mild cognitive impairment diagnosis	Article	2019	280
4	A convolutional neural network approach for classification of dementia stages based on 2D-spectral representation of EEG recordings	Article	2019	277
5	Automated multiclass classification of spontaneous EEG activity in Alzheimer’s disease and mild cognitive impairment	Article	2018	241
6	A new framework for automatic detection of patients with mild cognitive impairment using resting-state EEG signals	Article	2020	219
7	Spectral and complexity analysis of scalp EEG characteristics for mild cognitive impairment and early Alzheimer’s disease	Article	2014	218
8	EEGLAB: an open-source toolbox for analysis of single-trial EEG dynamics including independent component analysis	Article	2004	205
9	Systematic review on resting-state EEG for Alzheimer’s disease diagnosis and progression assessment	Review	2018	199
10	Combining EEG signal processing with supervised methods for Alzheimer’s patients’ classification	Article	2018	197
11	A single-channel EEG-based approach to detect mild cognitive impairment via speech-evoked brain responses	Article	2019	180
12	The diagnosis of mild cognitive impairment due to Alzheimer’s disease: recommendations from the National Institute on Aging-Alzheimer’s Association workgroups on diagnostic guidelines for Alzheimer’s disease	Article	2011	168
13	A long short-term memory-based framework for early detection of mild cognitive impairment from EEG signals	Article	2023	168
14	A comparison of resting state Alzheimer’s disease and mild EEG and structural MRI for classifying cognitive impairment	Article	2020	158
15	A novel electroencephalography-based approach for Alzheimer’s disease and mild cognitive impairment detection	Article	2021	157
16	Deep learning of resting-state electroencephalogram signals for three-class classification of Alzheimer’s disease, mild cognitive impairment and healthy ageing	Article	2021	155
17	A dementia classification framework using frequency and time-frequency features based on EEG signals	Article	2019	149
18	A novel methodology for automated differential diagnosis of mild cognitive impairment and Alzheimer’s disease using EEG signals	Article	2019	146
19	The diagnosis of dementia due to Alzheimer’s disease: recommendations from the National Institute on Aging-Alzheimer’s Association workgroups on diagnostic guidelines for Alzheimer’s disease	Article	2011	145
20	Early Alzheimer’s disease diagnosis based on EEG spectral images using deep learning	Article	2019	144

In terms of research content, optimization of EEG signal feature engineering, intelligent algorithm-assisted cognitive impairment classification, and exploration of multi-dimensional fusion schemes represent the current core research hotspots (Kashefpoor et al., 2016; Ruiz-Gómez et al., 2018; Sharma et al., 2019; Siuly et al., 2020).

### 3.5 Keyword co-occurrence analysis

Keyword co-occurrence analysis was conducted on the 523 included valid documents which were divided into two subsets: Traditional machine learning and deep learning. We screened out high-frequency keywords with a frequency of no less than 20 and sorted them in descending order of TLS. The results are summarized in Table 4.

**Table 4:** Comparison of keyword co-occurrence between traditional machine learning and deep learning literature (sorted by TLS in descending order).

Literature subset	Co-occurring keywords
Keyword co-occurrence in the traditional machine learning subset	Mild cognitive impairment, electroencephalography, Alzheimer disease, machine learning, support vector machine, cognitive defect, electroencephalogram, cognitive dysfunction, neurodegenerative diseases, mini mental state examination, sensitivity and specificity, EEG, feature extraction, cognitive impairment, cognition, diagnostic accuracy, functional connectivity, diagnosis, dementia, biological marker, nuclear magnetic resonance imaging, montreal cognitive assessment, random forest, algorithm, receiver operating characteristic, entropy, signal processing, feature selection, priority journal, Alzheimer’s disease, biomarkers, classification, artificial intelligence (*n* = 33)
Keyword co-occurrence in the deep learning literature	Electroencephalography, mild cognitive impairment, Alzheimer’s disease, deep learning, neurodegenerative diseases, cognitive impairment, convolutional neural network (CNN), cognitive defect, cognitive dysfunction, electroencephalogram, feature extraction, diagnosis, machine learning, neural networks, computer, dementia, early diagnosis, artificial neural network, Alzheimer’s disease, alzheimer, biomedical signal processing, support vector machine, functional connectivity, convolution, eeg, learning systems, classification (*n* = 26)

**Figure 6:**
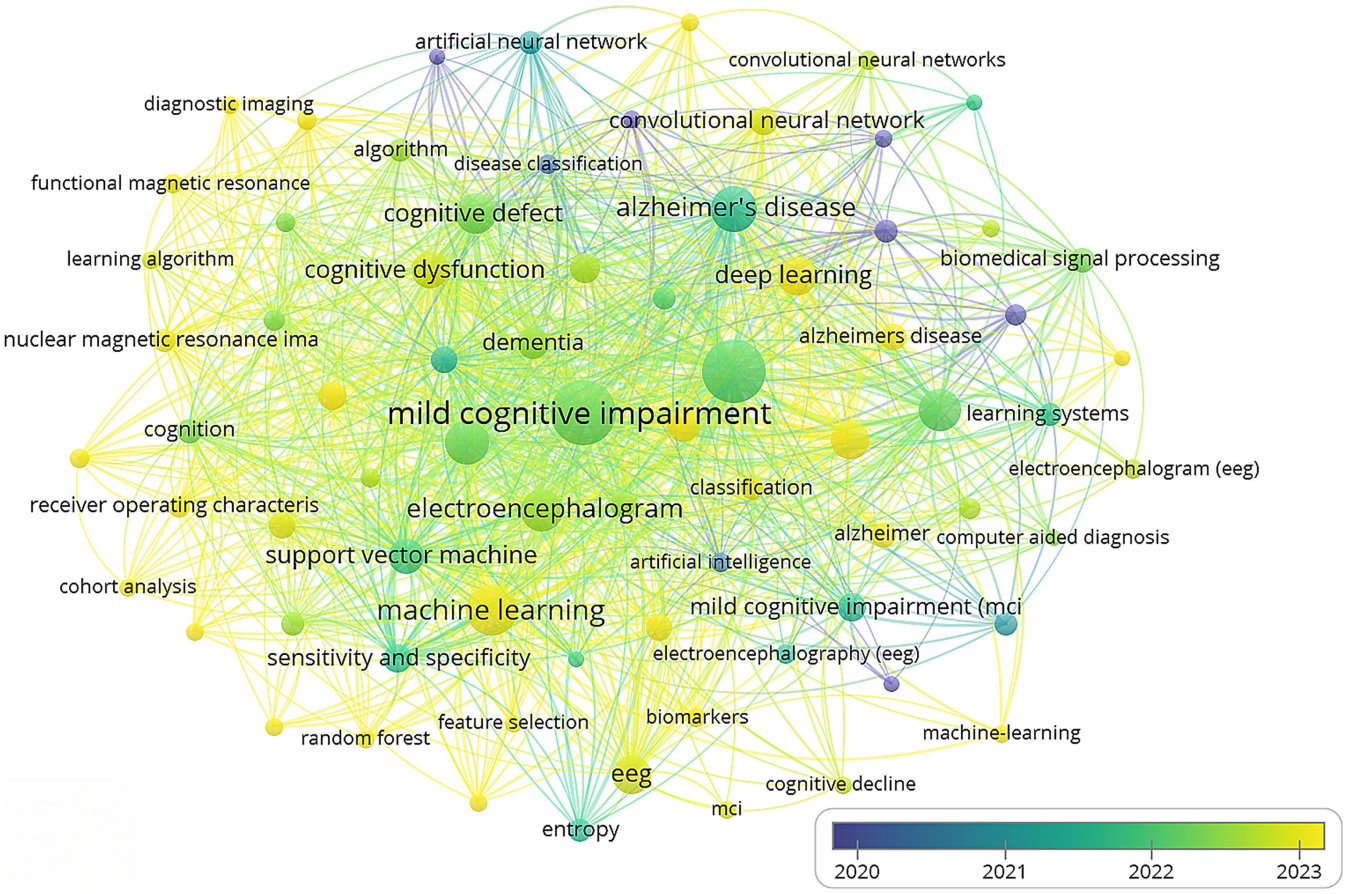
Keyword time-overlay visualization map.

Figure 6 shows the time-overlay visualization map of keywords. It summarizes the results of keyword co-occurrence analysis over time and clearly reveals the changing characteristics of research hotspots across different stages. The top 15 keywords sorted by frequency are: electroencephalography, mild cognitive impairment, Alzheimer’s disease, machine learning, cognitive defect, electroencephalogram, neurodegenerative diseases, cognitive dysfunction, cognitive impairment, support vector machine, Alzheimer’s disease, feature extraction, deep learning, diagnosis, sensitivity, and specificity.

**Figure 7:**
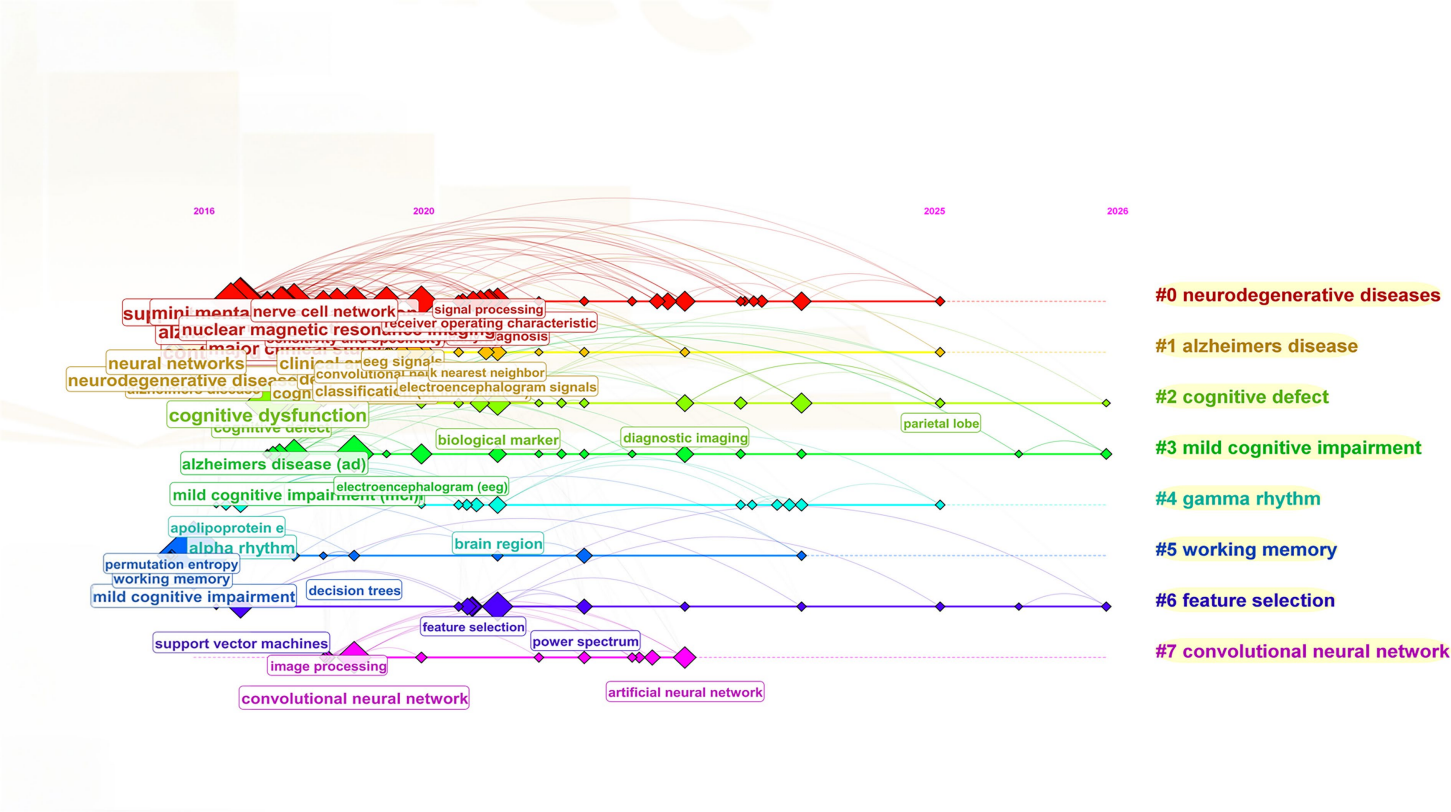
Keyword time-zone visualization map.

Figure 7 shows the time zone visualization of keywords. It further reveals the temporal evolution patterns of research themes. The results indicate that around 2016 was the initial formation stage of this research direction, with core keywords dominated by methodological themes, including permutation entropy, support vector machines, power spectrum, and alpha rhythm. From 2018 to 2020, the research focusses gradually shifted to clinical scenarios, and clinically oriented keywords such as mild cognitive impairment, Alzheimer’s disease, cognitive dysfunction, and biological markerbecame core clusters. Since 2020, themes such as feature selection and decision trees have emerged, and deep learning-related keywords including convolutional neural networks (CNNs) and artificial neural networks have begun to appear.

**Figure 8:**
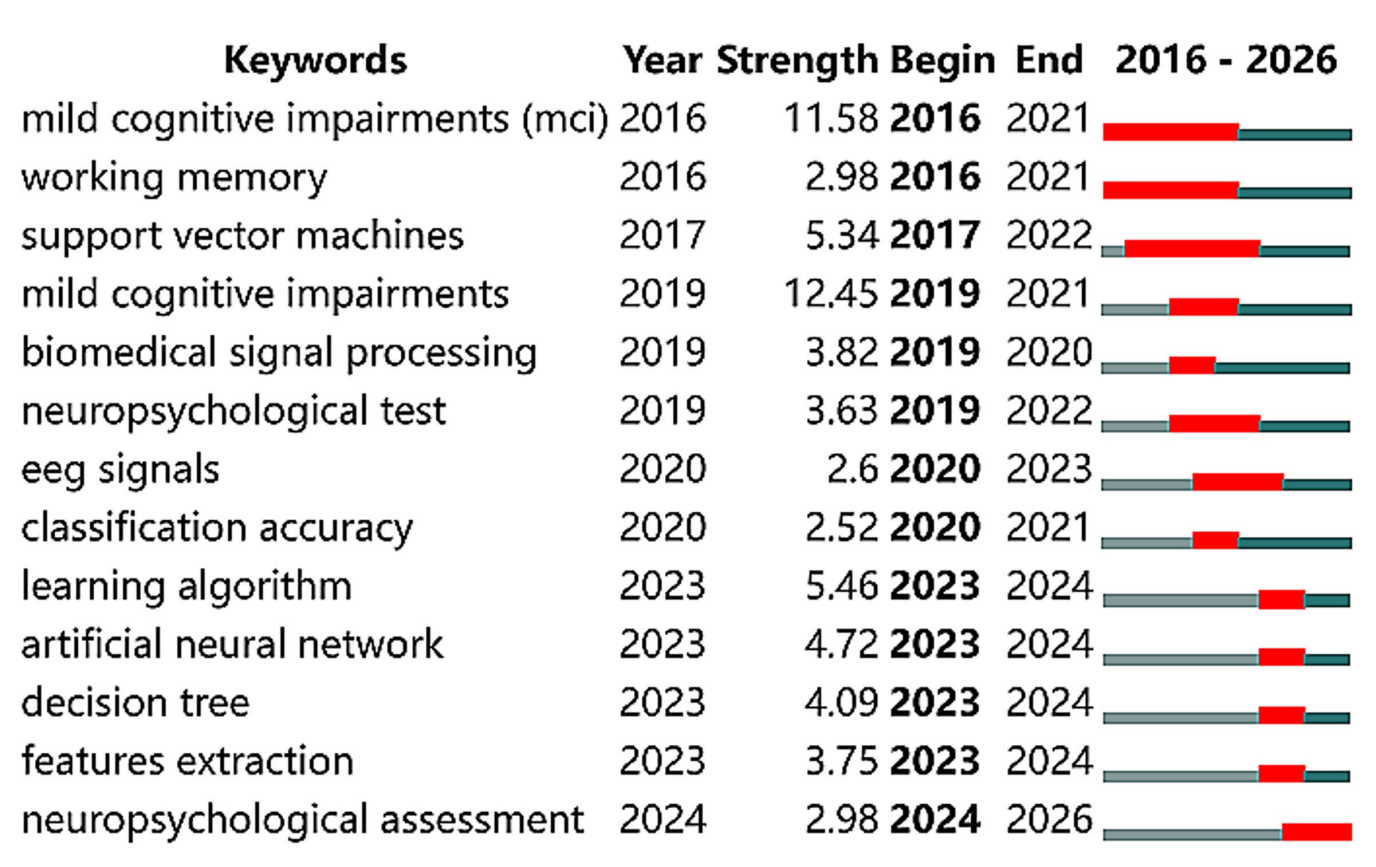
Top 13 keywords with the strongest citation bursts.

Keyword burst intensity is an important quantitative indicator. It measures research hotspots and emerging frontiers in the field at different periods. Figure 8 Shows that themes related to MCI have the highest burst intensity. In the early stages of research in this field support vector machines and working memory were representative hotspots. They were followed by themes such as biomedical signal processing EEG signals and classification accuracy. In recent years more attention has been paid to algorithms themselves including feature extraction decision tree and artificial neural network. Neuropsychological assessment is the only frontier theme that keeps bursting until 2026. Notably deep learning-related terms have not shown significant bursts. This may indicate that their application in this field is still in a stage of gradual growth and has not formed an explosive trend

**Figure 9:**
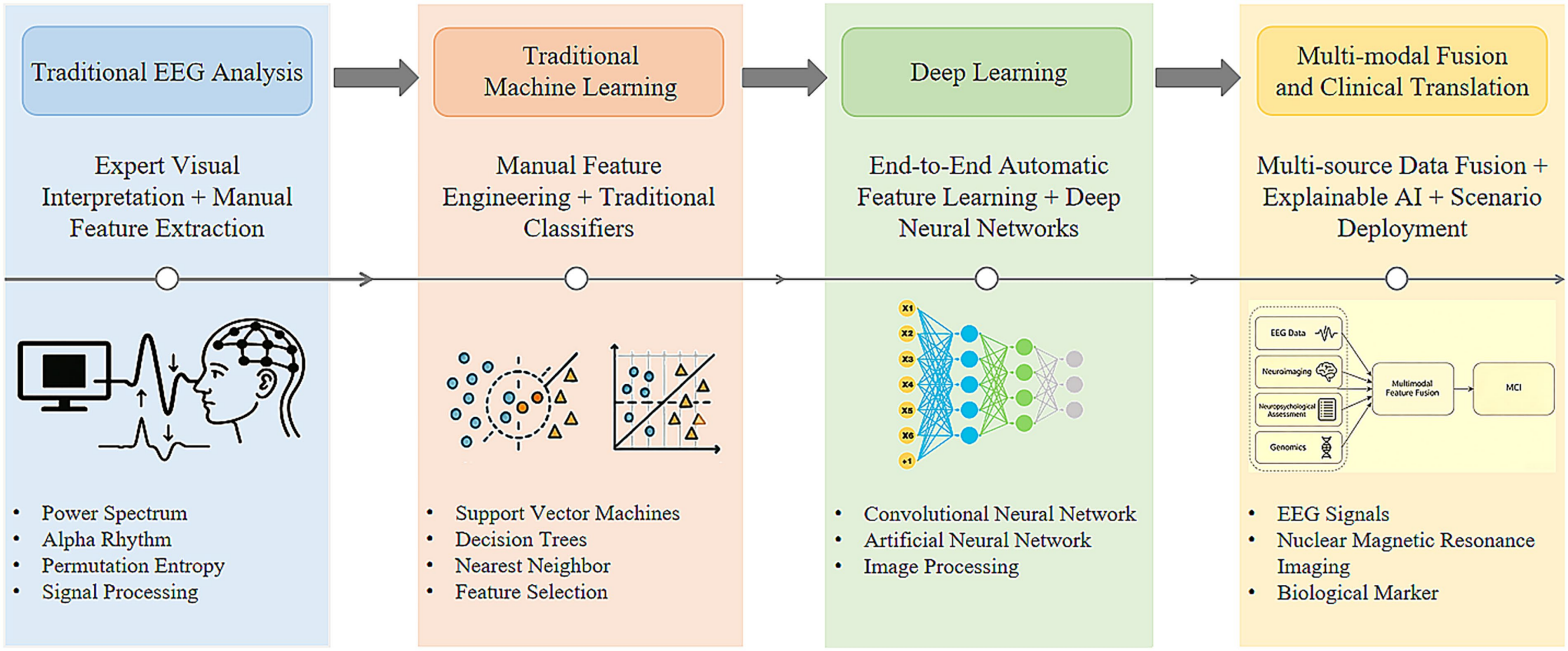
Technical evolution path of EEG-based MCI identification research.

Based on the above analysis of the keyword co-occurrence network, time-zone evolution patterns and burst themes, the technical evolution path of EEG-based MCI identification research can be summarized into four stages, as shown in Figure 9.

## 4 Discussion

### 4.1 Summary of key findings

This study integrated four major English databases (Web of Science, PubMed, IEEE Xplore, Scopus) and three major Chinese databases (CNKI, Wanfang, VIP). It conducted a bibliometric analysis of the relevant literature on the application of machine learning in EEG for MCI. The analysis covered publication trends, geographical distribution, core journals, intellectual foundations, and research hotspots.

The results show that the annual number of publications in this field exhibits a distinct three-stage growth pattern. The field entered a steady growth phase after 2018. The growth rate accelerated further after 2021. This trend may be driven by multiple potential factors. First, early studies mainly focused on applying and exploring traditional machine learning algorithms such as SVMs, gradually building the basic technical framework for EEG signal feature extraction and classification. With the development of deep learning technology, its end-to-end feature learning capability (Schirrmeister et al., 2017) has attracted extensive methodological optimization research in the academic community. Second, the intensification of global aging has highlighted the preventive and clinical value of MCI. MCI is the only modifiable prodromal stage of AD. Its annual dementia conversion rate of 10–15% (Livingston et al., 2020; Mitchell and Shiri-Feshki, 2009; Petersen et al., 2018) has generated an urgent demand for accurate, early diagnosis technologies. In addition, the widespread availability of multicenter open-access EEG databases after 2020 (Goldberger et al., 2000) has provided fundamental support for cross-institutional collaboration and large-sample studies.

In terms of national distribution, China ranks first worldwide, with 137 publications (26.2%). This output characteristic stems from two potential influencing factors. First, China has a large MCI patient population. The prevalence rate is 15.4% among those aged 55 years and older (Deng et al., 2021). Second, there is relevant policy guidance on the integrated development of artificial intelligence and healthcare in China (National Health Commission of China, 2025). It may also create a favorable external environment for research in this field. Nevertheless, it is worth noting that only 24 relevant studies were identified in China’s core Chinese databases. This represents a striking contrast to the high volume of international English publications. This discrepancy may be related to the long-standing emphasis on SCI/SSCI journals in China’s research evaluation system. Most high-quality research results are published in English for international academic exchange.

In terms of journal distribution, *Clinical Neurophysiology* serves as a core academic venue in this field. It has a TLS of 9,007. The top 10 highly co-cited journals cover multiple disciplines. These include neuroimaging, clinical neurophysiology, and geriatric neuroscience. This reflects the strong interdisciplinary nature of this field. Although China leads in publication volume, the United States still holds an advantage in the geographical distribution of journal hosting. Four of the top 10 highly co-cited journals are hosted by the U.S. Against the current development background of the field, the United States has established a relatively comprehensive research support system. Multicenter clinical repositories, represented by the Alzheimer’s Disease Neuroimaging Initiative (ADNI), provide high-quality longitudinal data (Beckett et al., 2015). Additionally, long-term funding from the U.S. BRAIN Initiative (Duncan et al., 2023; Miller et al., 2024) provides continuous financial support for the in-depth interdisciplinary integration of neuroscience, engineering, and artificial intelligence (Koroshetz et al., 2018).

### 4.2 Knowledge base

Highly cited articles form the core intellectual foundation of disciplinary development. The higher their citation frequency, the stronger their academic influence and core status in the field (Shen et al., 2022). In this study, the top 20 highly cited articles, ranked by TLS, can be divided into 4 core categories. Together, they construct a complete knowledge system of machine learning-based EEG in the field of MCI.

First, the literature on clinical diagnostic criteria for AD/MCI has established clinical normative foundation for relevant studies. Two core guidelines issued by the NIA-AA have clarified the diagnostic criteria and grading systems for Alzheimer’s disease dementia (McKhann et al., 2011) and MCI due to AD (Albert et al., 2011), respectively. They define the clinical definition and classification rules of MCI as the prodromal stage of AD. They provide a unified clinical basis for subject enrollment, disease staging, and clinical validation in various studies.

Second, studies on EEG electrophysiological characteristics in MCI/AD have provided a theoretical foundation for the application of EEG signals as potential biomarkers. Studies have confirmed that patients with MCI/AD exhibit characteristic resting-state EEG abnormalities. These include a low-frequency shift in the power spectrum, abnormal cortical rhythms, and decreased signal complexity (Fiscon et al., 2018; Cassani et al., 2018; Ruiz-Gómez et al., 2018). Furthermore, these electrophysiological changes are reported to be associated with the degree of cognitive decline, laying a potential pathophysiological foundation for EEG feature-based disease identification studies.

Third, the literature on EEG signal processing and feature engineering has established a methodological bridge between raw signals and effective feature extraction. The EEGLAB toolkit provides general tools for standardized preprocessing and independent component analysis of EEG data (Delorme and Makeig, 2004). Methods such as wavelet transform, spectral analysis, bispectral analysis, and non-linear dynamic features have become core techniques for feature extraction (Ieracitano et al., 2020). In addition, to adapt to the modeling needs of deep learning, strategies including the 2D image conversion method of EEG spectrum and multimodal feature fusion have also been proposed. According to published reports, these strategies have been shown to improve the representation capability of EEG features (Huggins et al., 2021; Ieracitano et al., 2019; Bi and Wang, 2019).

Finally, research on machine learning and deep learning classification models has developed a technical system for the intelligent diagnosis of MCI. Traditional machine learning algorithms, including SVM, multilayer perceptron, and k-nearest neighbor, are widely explored in EEG feature-based disease classification tasks. Related studies have reported favorable performance in both binary and multi-classification scenarios (Kashefpoor et al., 2016; Ruiz-Gómez et al., 2018; Sharma et al., 2019). Meanwhile, deep learning models such as CNN and Long Short-Term Memory have been extensively investigated for end-to-end feature learning and classification. Published studies have reported promising performance in multi-classification tasks of AD, MCI, and healthy controls (Alvi et al., 2023; Huggins et al., 2021), providing algorithmic references for technical iteration in EEG-based intelligent diagnosis research.

Overall, the above four categories of literature constitute the core knowledge base of this field, clarifying clinical diagnostic criteria and electrophysiological foundations, and establishing standardized technical tools and algorithmic frameworks. It provides comprehensive methodological support and research foundation for the subsequent research of EEG-based intelligent technologies in the early screening of MCI.

### 4.3 Research hotspots and trends

Through analysis of the keyword time-zone map and keyword burst map, the 20-year evolutionary trajectory of research hotspots in this field can be clearly delineated. The results show that the time-zone map identifies 8 core clusters: #0 Neurodegenerative Diseases, #1 Alzheimer’s Disease, #2 Cognitive Defect, #3 Mild Cognitive Impairment, #4 Gamma Rhythm, #5 Working Memory, #6 Feature Selection, #7 Convolutional Neural Network.

In the early research stage, core nodes were concentrated in #5 Working Memory and #7 Convolutional Neural Network. Core keywords include permutation entropy, alpha rhythm, support vector machines, and power spectrum. Studies at this stage mainly used non-linear dynamic features and basic EEG rhythms as core analytical indicators. They conducted exploratory research with traditional machine learning classifiers such as SVM (Sun et al., 2020). As the core cognitive domain earliest impaired in MCI, working memory serves as the primary reference indicator and research subject in early research (Kirova et al., 2015).

In the middle research stage, four disease-related clusters became the core hotspots. These include #0 Neurodegenerative Diseases, #1 Alzheimer’s Disease, #2 Cognitive Defect, and #3 Mild Cognitive Impairment. Clinically oriented keywords such as biological marker, diagnostic imaging, and electroencephalogram signals emerged intensively. Meanwhile, convolutional neural network in cluster #7 entered the research horizon. Deep learning began to develop in parallel with traditional machine learning. After preliminary feasibility was demonstrated in published studies, research quickly shifted from general cognitive impairment exploration to specific clinical diseases such as MCI and AD. Researchers were no longer confined to distinguishing general cognitive abnormalities. Instead, they focused on identifying specific diseases. The diagnostic value of EEG biomarkers became the core focus.

In the late research stage, cluster #6 Feature Selection emerged. Keywords including Decision Trees and Artificial Neural Network emerged intensively. Research began to focus on feature selection, multi-algorithm fusion, and improving model generalization. With the emergence of cluster #4 Gamma Rhythm, keywords including brain region, parietal lobe, and apolipoprotein E emerged intensively. Research deepened into more specific levels, covering refined EEG rhythm subdivision, brain region localization, and pathological correlation.

Based on the evolutionary trends of burst terms and clusters, three core development directions can be summarized as follows. First, the research focus is gradually shifting from merely pursuing peak classification accuracy to improving model generalization, enhancing algorithm interpretability, and optimizing adaptability to small-sample clinical data (Tokdar et al., 2026; Trinh et al., 2023). It aims to narrow the gap between high-performance laboratory outcomes and real-world clinical demands, and promote the iteration of algorithms from theoretical verification to clinical practice. Second, grouping and classification studies based on single EEG signals have gradually entered a bottleneck stage. Future research will better align with the real pathway of clinical diagnosis and evolve towards the multi-dimensional integration of EEG physiological indicators and neuropsychological scale assessment (Jiang et al., 2022). While retaining the advantages of objective EEG detection, this research direction incorporates clinical evaluation dimensions and is expected to improve the clinical reference value of relevant findings. Third, future research will further focus on subdivided EEG rhythms, brain region-specific alterations, and the exploration of pathology-associated biomarkers (Buzi et al., 2023; Ye et al., 2025; Yuan and Zhao, 2025). This will provide support for subsequent clinical translation and intervention exploration.

In addition, the application boundaries of the field are gradually expanding. On the one hand, research will extend from the core diseases of MCI and AD to cognitive impairment assessment in more neurodegenerative diseases (Falaschetti et al., 2024), exploring a universal cross-disease intelligent detection framework. On the other hand, with the combination of portable EEG devices and lightweight models (Nguyen-Quang et al., 2026), this technology is expected to extend to scenarios such as community screening, primary care, and home-based monitoring, thereby enhancing accessibility and broadening its application scope.

In contrast, relevant research in this field in China is constrained by the limited volume of literature. Studies have mostly focused on applying mature deep learning models such as CNNs and bidirectional long short-term memory networks. Moreover, technical approaches are limited to a single modality, electroencephalography. There is no in-depth exploration of cutting-edge directions including multimodal data fusion and innovative algorithm design. Clinical translation research lags behind, and no generalizable screening tool for primary care has been established yet.

### 4.4 Research limitations

This study has certain limitations. First, no systematic evaluation of the methodological quality of included original studies was conducted. In the field of cognitive impairment diagnosis using electroencephalography combined with artificial intelligence, the diagnostic performance of models is influenced by multiple key factors, including validation hierarchy, data leakage risk between training and test sets, EEG artifact preprocessing protocols, external independent dataset validation settings, sample size, class imbalance, and experimental reproducibility. The high-accuracy results widely reported in this field are often difficult to replicate in rigorous independent validation. As a bibliometric study, it did not perform stratified analyses of the above methodological heterogeneity. Therefore, trend interpretations based on publication volume and keywords can only reflect the academic community’s research attention and cannot be directly equated with the actual clinical efficacy of relevant technologies.

Second, the results of this study may be potentially affected by publication bias. Positive result publication bias is prevalent in this field: studies reporting positive results with high classification accuracy and significant performance improvements are more likely to be accepted by journals, while studies with negative results or non-significant performance gains face higher publication barriers. This publication tendency makes the research popularity and technological progress presented via bibliometric analysis in this study somewhat higher than the real development level of the field, which may interfere with the objective assessment of field trends.

Despite these limitations, the literature sample analysis based on seven databases in this study can still systematically and objectively reflect, to a certain extent, the overall research landscape, hotspot evolution patterns, and cutting-edge future trends of electroencephalography combined with machine learning and deep learning in the field of MCI. It can provide quantitative references and a decision-making basis for subsequent researchers to identify research directions and optimize experimental designs. It is hoped that future studies will conduct more in-depth bibliometric analysis combined with methodological quality evaluation and publication bias correction, to provide more comprehensive and rigorous data support for the development of the field.

## 5 Conclusion

In summary, bibliometric analysis indicates that research on electroencephalography-based MCI diagnosis with machine learning techniques has entered a rapid development stage. Research hotspots have gradually evolved in an iterative manner. Early research focused on exploring traditional machine learning algorithms and basic EEG feature analysis. Mid-term research gradually anchored to clinical disease entities such as MCI and AD. Current research is deepening toward directions including deep learning model optimization, feature selection and generalization improvement, and refined EEG rhythm and brain region localization. China ranks first globally in the number of publications in this field, while European and American countries still maintain advantages in academic discourse and access to core journals. Significant gaps remain between Chinese and English literature in research initiation time, overall output volume, and the depth of original exploration.

Future research should focus on three major directions. First, strengthen research on the interpretability and lightweight deployment of deep learning models. Combined with portable EEG devices, promote the application of such technologies in primary care and community screening scenarios. Second, integrate EEG with multidimensional information, including neuroimaging, neuropsychological assessment, and genomics, to construct more robust models for early diagnosis and progression prediction in MCI. Third, expand the application boundary of the technology by transferring existing analytical frameworks to cognitive impairment assessment in other neurodegenerative diseases, and explore a universal intelligent diagnostic system. The knowledge graph and trend analysis established in this study can provide a quantitative reference for researchers to identify suitable research entry points and conduct interdisciplinary collaboration, which is expected to provide a reference for the clinical translation of research in this field from bench to bedside.

## Data Availability

The original contributions presented in the study are included in the article/supplementary material, further inquiries can be directed to the corresponding author.

## References

[ref1] Aanestad E. Beniczky S. Olberg H. Brogger J. (2024). Unveiling variability: a systematic review of reproducibility in visual EEG analysis, with focus on seizures. Epileptic Disord. 26, 827–839. doi: 10.1002/epd2.20291 PMC11651379 39340408

[ref2] Albert M. S. DeKosky S. T. Dickson D. Dubois B. Feldman H. H. Fox N. C. . (2011). The diagnosis of mild cognitive impairment due to Alzheimer’s disease: recommendations from the national institute on aging-Alzheimer’s association workgroups on diagnostic guidelines for Alzheimer’s disease. Alzheimers Dement. 7, 270–279. doi: 10.1016/j.jalz.2011.03.008, 21514249 PMC3312027

[ref3] Alvi A. M. Siuly S. Wang H. (2023). A long short-term memory based framework for early detection of mild cognitive impairment from EEG signals. IEEE Trans. Emerg. Top. Comput. Intell. 7, 375–388. doi: 10.1109/TETCI.2022.3186180

[ref4] Beckett L. A. Donohue M. C. Wang C. Aisen P. Harvey D. J. Saito N. . (2015). The Alzheimer’s disease neuroimaging initiative phase 2: increasing the length, breadth, and depth of our understanding. Alzheimers Dement. 11, 823–831. doi: 10.1016/j.jalz.2015.05.004, 26194315 PMC4510463

[ref5] Bi X. Wang H. (2019). Early Alzheimer’s disease diagnosis based on EEG spectral images using deep learning. Neural Netw. 114, 119–135. doi: 10.1016/j.neunet.2019.02.005 30903945

[ref6] Bradfield N. I. (2023). Mild cognitive impairment: diagnosis and subtypes. Clin. EEG Neurosci. 54, 4–11. doi: 10.1177/15500594211042708, 34549629

[ref7] Buzi G. Fornari C. Perinelli A. Mazza V. (2023). Functional connectivity changes in mild cognitive impairment: a meta-analysis of M/EEG studies. Clin. Neurophysiol. 156, 183–195. doi: 10.1016/j.clinph.2023.10.011 37967512

[ref8] Cassani R. Estarellas M. San-Martin R. Fraga F. J. Falk T. H. (2018). Systematic review on resting-state EEG for Alzheimer’s disease diagnosis and progression assessment. Dis. Markers 2018:5174815. doi: 10.1155/2018/5174815 PMC6200063 30405860

[ref9] Chen D. Zhang G. Wang J. Chen S. Wang J. Nie H. . (2021). Mapping trends in moyamoya angiopathy research: A 10-year bibliometric and visualization-based analyses of the web of science core collection (WoSCC). Front. Neurol. 12:637310. doi: 10.3389/fneur.2021.637310, 33737903 PMC7960774

[ref10] Chriskos P. Neophytou K. Frantzidis C. A. Gallegos J. Afthinos A. Onyike C. U. . (2025). The use of low-density EEG for the classification of PPA and MCI. Front. Hum. Neurosci. 19:1526554. doi: 10.3389/fnhum.2025.1526554, 39989721 PMC11842309

[ref11] Craik A. He Y. Contreras-Vidal J. L. (2019). Deep learning for electroencephalogram (EEG) classification tasks: a review. J. Neural Eng. 16:031001. doi: 10.1088/1741-2552/ab0ab5, 30808014

[ref12] Delorme A. Makeig S. (2004). EEGLAB: an open source toolbox for analysis of single-trial EEG dynamics including independent component analysis. J. Neurosci. Methods 134, 9–21. doi: 10.1016/j.jneumeth.2003.10.009, 15102499

[ref13] Deng Y. Zhao S. Cheng G. Yang J. Li B. Xu K. . (2021). The prevalence of mild cognitive impairment among Chinese people: a meta-analysis. Neuroepidemiology 55, 79–91. doi: 10.1159/000512597, 33756479

[ref14] Donthu N. Kumar S. Mukherjee D. Pandey N. Lim W. M. (2021). How to conduct a bibliometric analysis: an overview and guidelines. J. Bus. Res. 133, 285–296. doi: 10.1016/j.jbusres.2021.04.070

[ref15] Duncan D. Garner R. Brinkerhoff S. Walker H. C. Pouratian N. Toga A. W. (2023). Data archive for the BRAIN initiative (DABI). Sci. Data 10:83. doi: 10.1038/s41597-023-01972-z, 36759619 PMC9911697

[ref16] Falaschetti L. Biagetti G. Alessandrini M. Turchetti C. Luzzi S. Crippa P. (2024). Multi-class detection of neurodegenerative diseases from EEG signals using lightweight LSTM neural networks. Sensors (Basel, Switzerland) 24:6721. doi: 10.3390/s24206721, 39460201 PMC11511166

[ref17] Fiscon G. Weitschek E. Cialini A. Felici G. Bertolazzi P. De Salvo S. . (2018). Combining EEG signal processing with supervised methods for Alzheimer’s patients classification. BMC Med. Inform. Decis. Mak. 18:35. doi: 10.1186/s12911-018-0613-y, 29855305 PMC5984382

[ref18] Franzmeier N. Göttler J. Grimmer T. Drzezga A. Áraque-Caballero M. A. Simon-Vermot L. . (2017). Resting-state connectivity of the left frontal cortex to the default mode and dorsal attention network supports reserve in mild cognitive impairment. Front. Aging Neurosci. 9:264. doi: 10.3389/fnagi.2017.00264, 28824423 PMC5545597

[ref19] Goldberger A. L. Amaral L. A. N. Glass L. Hausdorff J. M. Ivanov P. C. Mark R. G. . (2000). PhysioBank, PhysioToolkit, and PhysioNet. Circulation 101, e215–e220. doi: 10.1161/01.CIR.101.23.e215, 10851218

[ref20] Huggins C. J. Escudero J. Parra M. A. Scally B. Anghinah R. Vitória Lacerda De Araújo A. . (2021). Deep learning of resting-state electroencephalogram signals for three-class classification of Alzheimer’s disease, mild cognitive impairment and healthy ageing. J. Neural Eng. 18:046087. doi: 10.1088/1741-2552/ac05d8 34044374

[ref21] Ieracitano C. Mammone N. Bramanti A. Hussain A. Morabito F. C. (2019). A convolutional neural network approach for classification of dementia stages based on 2D-spectral representation of EEG recordings. Neurocomputing 323, 96–107. doi: 10.1016/j.neucom.2018.09.071

[ref22] Ieracitano C. Mammone N. Hussain A. Morabito F. C. (2020). A novel multi-modal machine learning based approach for automatic classification of EEG recordings in dementia. Neural Netw. 123, 176–190. doi: 10.1016/j.neunet.2019.12.006, 31884180

[ref23] Jiang Y. Guo Z. Jiao R. He H. Jiang N. He J. (2025). Abnormal resting-state EEG neural oscillations and functional connectivity in mild cognitive impairment. Front. Aging Neurosci. 17:1640966. doi: 10.3389/fnagi.2025.1640966, 41019423 PMC12463927

[ref24] Jiang J. Zhang J. Li C. Yu Z. Yan Z. Jiang J. (2022). Development of a machine learning model to discriminate mild cognitive impairment subjects from normal controls in community screening. Brain Sci. 12:1149. doi: 10.3390/brainsci12091149 PMC9497124 36138886

[ref25] Jongsiriyanyong S. Limpawattana P. (2018). Mild cognitive impairment in clinical practice: a review article. Am. J. Alzheimers Dis. Other Dement. 33, 500–507. doi: 10.1177/1533317518791401, 30068225 PMC10852498

[ref26] Kashefpoor M. Rabbani H. Barekatain M. (2016). Automatic diagnosis of mild cognitive impairment using electroencephalogram spectral features. J. Med. Signals Sensors 6, 25–32. doi: 10.4103/2228-7477.175869 PMC4786960 27014609

[ref27] Kirova A. M. Bays R. B. Lagalwar S. (2015). Working memory and executive function decline across normal aging, mild cognitive impairment, and Alzheimer’s disease. Biomed. Res. Int. 2015:748212. doi: 10.1155/2015/748212 PMC4624908 26550575

[ref28] Koroshetz W. Gordon J. Adams A. Beckel-Mitchener A. Churchill J. Farber G. . (2018). The state of the NIH BRAIN initiative. J. Neurosci. 38, 6427–6438. doi: 10.1523/JNEUROSCI.3174-17.2018, 29921715 PMC6052245

[ref29] Livingston G. Huntley J. Sommerlad A. Ames D. Ballard C. Banerjee S. . (2020). Dementia prevention, intervention, and care: 2020 report of the lancet commission. Lancet 396, 413–446. doi: 10.1016/S0140-6736(20)30367-6, 32738937 PMC7392084

[ref30] McKhann G. M. Knopman D. S. Chertkow H. Hyman B. T. Jack C. R. Kawas C. H. . (2011). The diagnosis of dementia due to Alzheimer’s disease: recommendations from the national institute on aging-Alzheimer’s association workgroups on diagnostic guidelines for Alzheimer’s disease. Alzheimers Dement. 7, 263–269. doi: 10.1016/j.jalz.2011.03.005, 21514250 PMC3312024

[ref31] Miller C. T. Chen X. Donaldson Z. R. Marlin B. J. Tsao D. Y. Williams Z. M. . (2024). The BRAIN initiative: a pioneering program on the precipice. Nat. Neurosci. 27, 2264–2266. doi: 10.1038/s41593-024-01811-3 39578571

[ref32] Mitchell A. J. Shiri-Feshki M. (2009). Rate of progression of mild cognitive impairment to dementia – meta-analysis of 41 robust inception cohort studies. Acta Psychiatr. Scand. 119, 252–265. doi: 10.1111/j.1600-0447.2008.01326.x, 19236314

[ref33] National Health Commission of China (2025). Implementation guidelines on promoting and regulating the development of "AI in healthcare" applications. Med. Inf. 38:193. (in Chinese)

[ref34] Nguyen-Quang T. Ngo M. L. Nguyen N.-S. Vinh N. T. Han H.-D. Tung B. T. . (2026). DeapTokenEEG enhancing mild cognitive impairment and Alzheimer's classification via tokenized EEG features. arXiv. [Preprint]. arXiv:2605.15009v1. Available online at: https://arxiv.org/abs/2605.15009 (Accessed June 20, 2026).

[ref35] Petersen R. C. Lopez O. Armstrong M. J. Getchius T. S. D. Ganguli M. Gloss D. . (2018). Practice guideline update summary: mild cognitive impairment [RETIRED]: report of the guideline development, dissemination, and implementation subcommittee of the American academy of neurology. Neurology 90, 126–135. doi: 10.1212/WNL.0000000000004826, 29282327 PMC5772157

[ref36] Ruiz-Gómez S. J. Gómez C. Poza J. Gutiérrez-Tobal G. C. Tola-Arribas M. A. Cano M. . (2018). Automated multiclass classification of spontaneous EEG activity in Alzheimer’s disease and mild cognitive impairment. Entropy 20:35. doi: 10.3390/e20010035, 33265122 PMC7512207

[ref37] Scheltens P. Strooper B. D. Kivipelto M. Holstege H. Chételat G. Teunissen C. E. . (2021). Alzheimer’s disease. Lancet 397, 1577–1590. doi: 10.1016/S0140-6736(20)32205-4, 33667416 PMC8354300

[ref38] Schirrmeister R. T. Springenberg J. T. Fiederer L. D. J. Glasstetter M. Eggensperger K. Tangermann M. . (2017). Deep learning with convolutional neural networks for EEG decoding and visualization. Hum. Brain Mapp. 38, 5391–5420. doi: 10.1002/hbm.23730, 28782865 PMC5655781

[ref39] Sharma N. Kolekar M. H. Jha K. Kumar Y. (2019). EEG and cognitive biomarkers based mild cognitive impairment diagnosis. IRBM 40, 113–121. doi: 10.1016/j.irbm.2018.11.007

[ref40] Shen J. Shen H. Ke L. Chen J. Dang X. Liu B. . (2022). Knowledge mapping of immunotherapy for hepatocellular carcinoma: a bibliometric study. Front. Immunol. 13:815575. doi: 10.3389/fimmu.2022.815575, 35173728 PMC8841606

[ref41] Siuly S. Alcin O. F. Kabir E. Sengur A. Wang H. Zhang Y. . (2020). A new framework for automatic detection of patients with mild cognitive impairment using resting-state EEG signals. IEEE Trans. Neural Syst. Rehabil. Eng. 28, 1966–1976. doi: 10.1109/TNSRE.2020.3013429, 32746328

[ref42] Sun J. Wang B. Niu Y. Tan Y. Fan C. Zhang N. . (2020). Complexity analysis of EEG, MEG, and fMRI in mild cognitive impairment and Alzheimer’s disease: a review. Entropy (Basel, Switzerland) 22:239. doi: 10.3390/e22020239 PMC7516672 33286013

[ref43] Tokdar A. Agarwal L. Chatterjee S. Sukriti (2026). A deep hybrid CNN-BiLSTM-BiGRU architecture with explainability for mild cognitive impairment detection using EEG. Brain Inform. 13:17. doi: 10.1186/s40708-026-00302-4 PMC13194862 42108320

[ref44] Trinh T.-T. Liu Y.-H. Wu C.-T. Peng W.-H. Hou C.-L. Weng C.-H. . (2023). PLI-based connectivity in resting-EEG is a robust and Generalizable feature for detecting MCI and AD: a validation on a diverse Multisite Clinical Dataset. Ann. Int. Conf. IEEE Eng. Med. Biol. Soc. 2023, 1–6. doi: 10.1109/EMBC40787.2023.10340854 38083569

[ref45] Vega J. N. Newhouse P. A. (2014). Mild cognitive impairment: diagnosis, longitudinal course, and emerging treatments. Curr. Psychiatry Rep. 16:490. doi: 10.1007/s11920-014-0490-8, 25160795 PMC4169219

[ref46] Wijaya A. Setiawan N. A. Ahmad A. H. Zakaria R. Othman Z. (2023). Electroencephalography and mild cognitive impairment research: a scoping review and bibliometric analysis (ScoRBA). AIMS Neurosci. 10, 154–171. doi: 10.3934/Neuroscience.2023012, 37426780 PMC10323261

[ref47] Ye X. Yan Y. Wang Y. Shi J. (2025). EEG-based minimum spanning tree analysis reveals network disruptions in Alzheimer’s disease spectrum: an observational study. Front. Aging Neurosci. 17:1604345. doi: 10.3389/fnagi.2025.1604345 PMC12511049 41079997

[ref48] Yuan Y. Zhao Y. (2025). The role of quantitative EEG biomarkers in Alzheimer’s disease and mild cognitive impairment: applications and insights. Front. Aging Neurosci. 17:1522552. doi: 10.3389/fnagi.2025.1522552 PMC12055827 40336944

